# Laser Catheter Modulation of the Sinus Node in the Treatment of Inappropriate Sinus Tachycardia: Experimental and Clinical Results

**DOI:** 10.19102/icrm.2018.090704

**Published:** 2018-07-15

**Authors:** Helmut P. Weber, Armin Heinze, Lutz Ruprecht, Michaela Sagerer-Gerhardt

**Affiliations:** ^1^Lasers and Applied Technologies Laboratory, Hospital Harlaching, Teaching Hospital LM-University of Munich, Munich, Germany; ^2^Department of Research Development Education, CCEP Center Taufkirchen, Taufkirchen, Germany; ^3^Laboratory Animal Facilities, Helmholtz Center Munich, Neuherberg, Germany; ^4^Department of Anesthesiology, Hospital Neuperlach, Teaching Hospital LM-University of Munich, Munich, Germany

**Keywords:** Focused local electrogram, inappropriate sinus tachycardia, laser catheter ablation, monitoring of local electrical potential amplitudes

## Abstract

Inappropriate sinus tachycardia (IST) is a rare type of arrhythmia that is currently difficult to treat successfully. The effects of laser catheter applications aimed at the sinus nodal area were tested experimentally and the technique was used for the treatment of IST. Continuous-wave, mapping-guided 1,064 nm neodymium-doped yttrium aluminum garnet laser applications at 15 W (9.5 W/mm^2^) per 15 seconds (142.5 J/mm^2^) and an irrigation flow of 30 mL/min were aimed at the sinus nodal area in five dogs (three applications each) and one human patient (two applications) by use of an 8-French open-irrigated electrode-laser mapping and ablation (ELMA) catheter provided with three miniature pin electrodes (0.5 mm × 4.0 mm) with interelectrode distances of 2.0 mm arranged symmetrically and radially around the endhole of the catheter tip. Laser application was aimed at the largest and earliest atrial potentials recorded in the focused local electrograms 30 ms to 45 ms prior to the onset of the P-wave in the surface lead electrocardiogram. Lesions were evaluated morphometrically. Holter monitoring in the patient was performed prior to and after treatment. During laser application in the dogs, sinus nodal potential amplitudes dwindled gradually from a mean of 42 mm ± 24 mm to 5.0 mm ± 3.0 mm and sinus cycle lengths lengthened from 452 ms ± 35 ms to 634 ms ± 35 ms (p < 0.0001 for both). In the patient, electrical potential amplitudes in the local electrograms dwindled from 41.0 mm to 5.0 mm and, in the Holter monitor, heart rate decreased from 109 bpm ± 29 bpm to 79 bpm ± 26 bpm (p < 0.0001). IST ablation was painless and without complications. During a follow-up of 4.9 years, the patient was asymptomatic and her heart rate and chronotropic competence remained normal. In conclusion, ablation of IST was achieved by substrate mapping-guided laser application while using the open-irrigated EMLA catheter RytmoLas (LasCor GmbH – Laser Medical Devices, Taufkirchen, Germany). However, this is a proof-of-concept study and further research, preferably in the form of multicenter study trials, is needed for confirmation of the results.

## Introduction

Inappropriate sinus tachycardia (IST), first described in 1979,^1^ is a rare condition in which the resting heart rate is abnormally high: specifically, the average heart rate is usually > 95 bpm and can rapidly accelerate to more than 100 bpm without an identifiable cause, although exercise and emotional stress have been identified as triggering factors. IST is a more long-standing problem with unknown causes and electrocardiograms (ECGs) that do not show any abnormalities; some research has suggested it may be triggered by the sinus node itself having an abnormal structure or function, or it may be the result of a failure of the autonomic nervous systems. Immunologic disorders have also been reported.^2^ IST does not predict higher rates of mortality and is viewed by most to be a benign condition in the long-term; thus, nontreatment is an option chosen by many, if symptoms are minimal. However, in some patients, symptoms may be distracting and warrant treatment. In these people, monitoring the condition is recommended.^3^ The diagnosis of IST is primarily one involving the exclusion of all other causes of sinus tachycardia and common forms of supraventricular tachycardia. A mean heart rate of > 95 bpm and inappropriate heart rate response on exertion are usually but not always present. In symptomatic patients, IST has been treated both pharmacologically and invasively, with varying degrees of success. Types of medication tried include b-blockers, calcium channel blockers, and—more recently—a new selective sinus node inhibitor called ivabradin.^4^ Invasive treatments include forms of radiofrequency catheter ablation such as sinus node modification,^5^ complete sinus node ablation, and atrioventricular node ablation in very resistant cases, with associated implantation of a permanent artificial pacemaker. However, it is currently difficult to treat IST successfully, and invasive treatments can also make the symptoms worse.^3^ In the following, we present details on attempted sinus nodal modulation in a dog model and a human patient with IST by using transcatheter continuous-wave laser application guided by the monitoring of electrical potential amplitudes in the focused local electrograms (LEGs) recorded via the pin electrodes of an electrode-laser mapping and ablation (ELMA) catheter with narrow interelectrode distances.

## Methods and results

**Figure 1:**
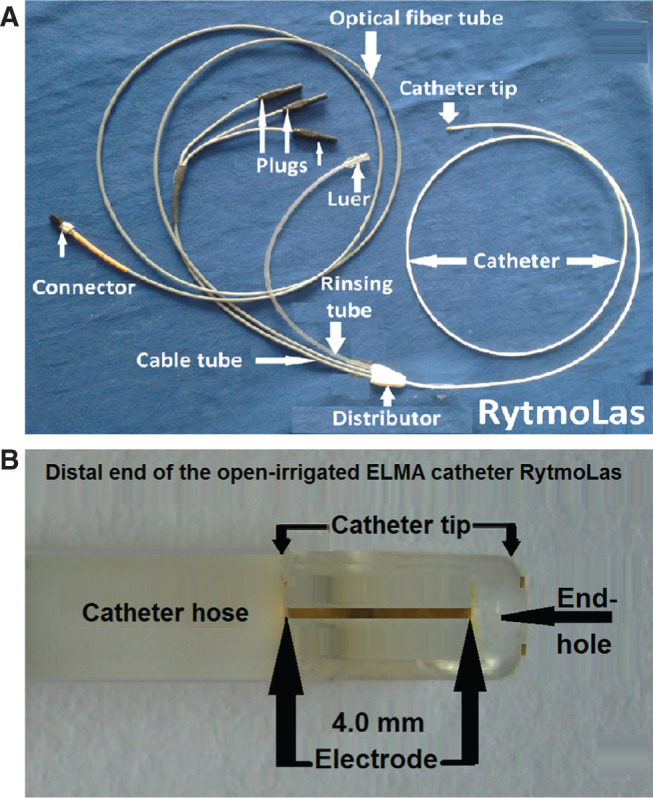
**A:** An overview of the ELMA catheter RytmoLas (LasCor GmbH – Laser Medical Devices, Taufkirchen, Germany). **B:** Distal segment of the open-irrigated ELMA catheter RytmoLas (LasCor GmbH – Laser Medical Devices, Taufkirchen, Germany) showing the tip of the catheter with the cable electrodes mounted longitudinally and symmetrically on the outer surface of the tip at distances of ≤ 2.0 mm from one another.

**Figure 2:**
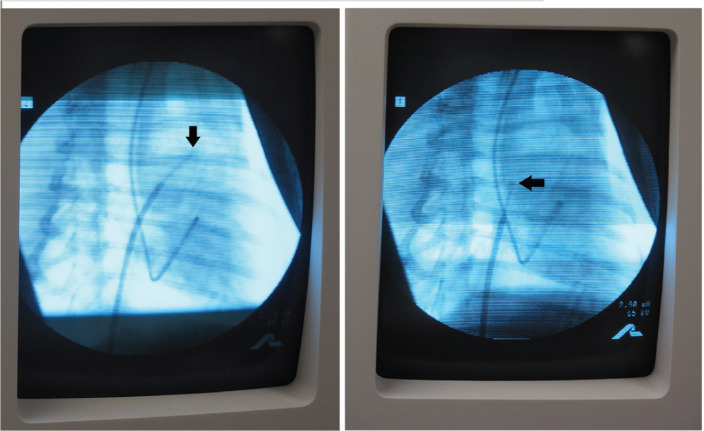
Right anterior oblique X-ray images of a dog heart during catheter mapping of the high right atrium and the assumed sinus nodal area for localization of the earliest atrial potential with the highest amplitude in the focused local electrogram. The image shows the open-irrigated ELMA catheter RytmoLas (LasCor GmbH – Laser Medical Devices, Taufkirchen, Germany) with its tip (black arrows) moved from the anterior region of the right atrial roof (left, vertical arrow) to a more posterolateral region (right, horizontal arrow). The catheter was fed into a steerable Agilis™ sheath (Abbott Laboratories, Chicago, IL, USA) introduced perivenously from the groin. The second catheter is a multipolar ring-electrode catheter positioned in the coronary sinus.

The laser used was a 1,064-nm diode laser (MediLas; LasCor GmbH – Laser Medical Devices, Taufkirchen, Germany) with a maximum power of 30 W that was provided with a roller pump with 5 mL to 60 mL of saline flow per minute and a footswitch. The open-irrigated ELMA catheter used (RytmoLas; LasCor GmbH – Laser Medical Devices, Taufkirchen, Germany) was an 8-French (Fr) tripolar catheter with an optical fiber fed into its central lumen and mounted in front of the catheter endhole. The catheter tip included three small (9.5 mm × 4.0 mm) pin electrodes with interelectrode distances of 2.0 mm apart arranged symmetrically around the rim of the catheter endhole **(Figure 1)**. The electrodes were connected to the manifold via cables running in the catheter lumen. The proximal end of the optical fiber was provided with a connector for the laser. The catheter was flushed continuously with saline via an irrigation line at a rate of 15 mL/min, which was increased automatically to 30 mL/min by the foot switch when the laser was activated. The development of the open-irrigated ELMA catheter is described in detail elsewhere.^6^ Percutaneous access to the femoral vein was obtained using the Seldinger technique. Once access was secured, a steerable sheath (Agilis™; Abbott Laboratories, Chicago, IL, USA) was advanced to the level of the right atrium. The catheter was introduced through this sheath and was manipulated under X-ray control **(Figure 2)** towards the sinus nodal area, where the earliest and largest electrical potential amplitudes were recorded from. Under continuous control of local electrical potential amplitudes in the focused LEGs, mapping-guided continuous-wave 1,064 nm neodymium-doped yttrium aluminum garnet laser applications at 15 W (9.5 W/mm^2^) per 15 seconds (142.5 J/mm^2^) were aimed selectively at these areas.

### Animal experiments

The animal investigations complied with the principles outlined in the Declaration of Helsinki. All animal experimental studies conformed to “Directive 86/609/EEC on the Protection of Animals Used for Experimental and Other Scientific Purposes,” which was adopted in 1986 by the European Commission.

**Figure 3:**
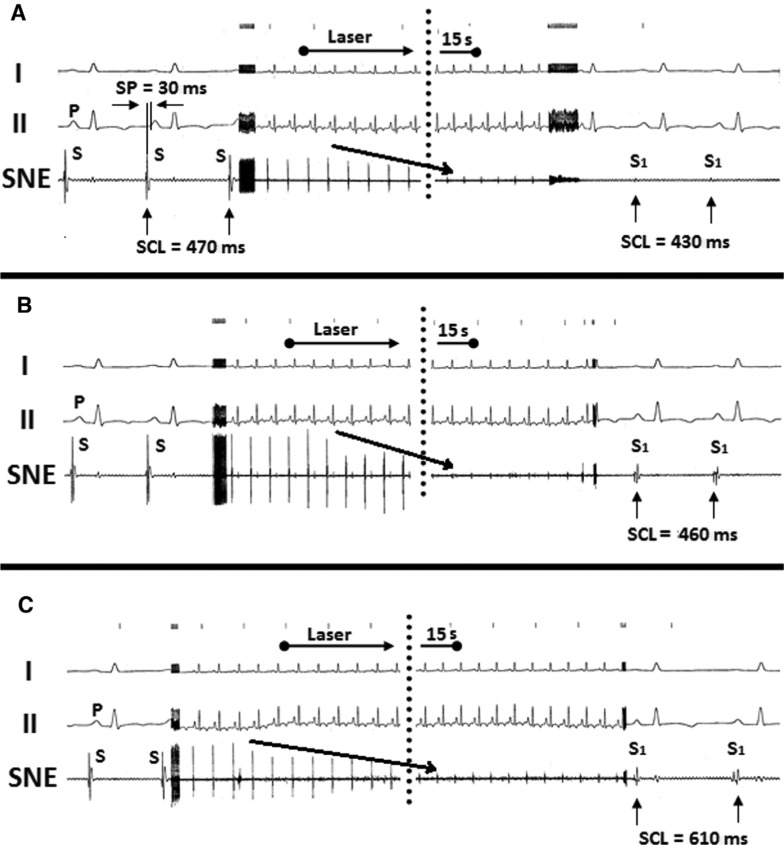
Bipolar focused local electrograms recorded via the small pin electrodes with 2.0 mm interelectrode distances mounted at the tip of the open-irrigated ELMA catheter RytmoLas (LasCor GmbH – Laser Medical Devices, Taufkirchen, Germany). This image presents three laser applications at time intervals of five minutes each, all aimed at the sinus nodal area showing the earliest atrial activation (SP) equal to between 30 ms and 35 ms prior to the onset of the P-waves in the surface lead electrograms I and II. During laser application, electrical potential amplitudes (S) gradually dwindle and, after 15 seconds of radiation, are practically abolished (S1). In **A** and **B**, the SCLs are shortening, while, in **C**, the SCL is lengthening. SNE: sinus nodal electrogram.

**Figure 4:**
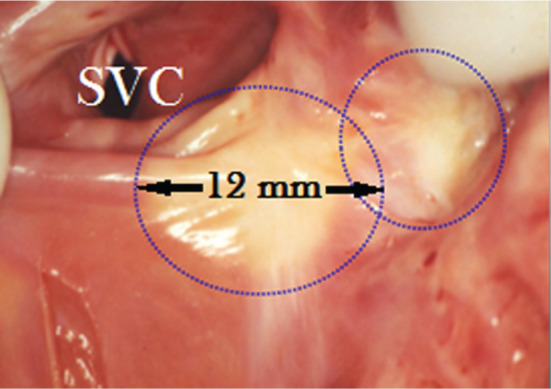
Endocardial view of the sinus nodal area of a dog three months after laser applications performed at 15 W for 15 seconds aimed at the region wherein the earliest electrical activation was localized during catheter mapping. Clear-cut pale fibrous scars (circles) without aneurysm formation and without signs of tissue vaporization with crater formation can be seen. SVC: superior vena cava.

Laser catheter applications were aimed at the sinus nodal regions in five anesthetized dogs, with each dog receiving three applications each. During laser application, the amplitudes of atrial potentials decreased gradually from in the range of 15 mm to 44 mm (42 mm ± 24 mm) to in that of 0 mm to 11 mm (5.0 mm ± 3.0 mm) (p < 0.0001) and, after 15 seconds of radiation, were abolished entirely. In addition, sinus cycle length (SCL) was prolonged from between 410 ms and 490 ms (452 ms ± 35 ms) to between 590 ms and 690 ms (634 ms ± 35 ms) (p < 0.0001). In some cases, the first two applications shortened sinus SCL but, following the third application, SCL increased **(Figure 3)**. The interventions were completed without complications and weekly electrograms performed during the follow-up period of three months showed permanent reduced heart rates as compared with those recorded prior to the laser experiments (mean: 95 bpm ± 9.3 bpm versus mean: 79 bpm ± 4.2 bpm; p = 0.008). The dogs were euthanized after three months, at which point gross pathology of the irradiated area showed contiguous transmural, clear-cut circular dense fibrous scars without signs of aneurysm or crater formation **(Figure 4)**.

### Inappropriate sinus tachycardia ablation

A 38-year-old female patient was referred to our hospital for the ablation of IST. Attempted radiofrequency IST ablation one year prior had been unsuccessful. Laser ablation was granted a favorable ethical opinion and was approved by the Ethics Committee of the Board of Physicians of the Land of Bavaria (reference EK/h no: 95243). Written informed consent was obtained from the patient 24 hours prior to the procedure.

The patient suffered from attacks of palpitation, anxiety, panic, breathlessness, and angina despite medication with 80 mg of sotalol three times daily and 0.2 mg of β-acetyl digoxin per day. The attacks were triggered mainly by emotional stress but also occurred spontaneously while the patient was at rest. The patient had no other active medical problems and was otherwise well. Her vital signs were a blood pressure of 110/65 mmHg and a pulse of 75 bpm. Due to history and previous medical records indicating postural tachycardia syndrome, other potential causes of tachycardia were excluded. Her routine laboratory findings including thyroid function were normal, while her digoxin serum level was 0.55 ng/mL. The patient refused to discontinue medication and to stay in the hospital. She received a Holter monitor, gave written informed consent for an invasive electrophysiology (EP) study with optional laser ablation for IST, and left the clinic.

**Figure 5:**
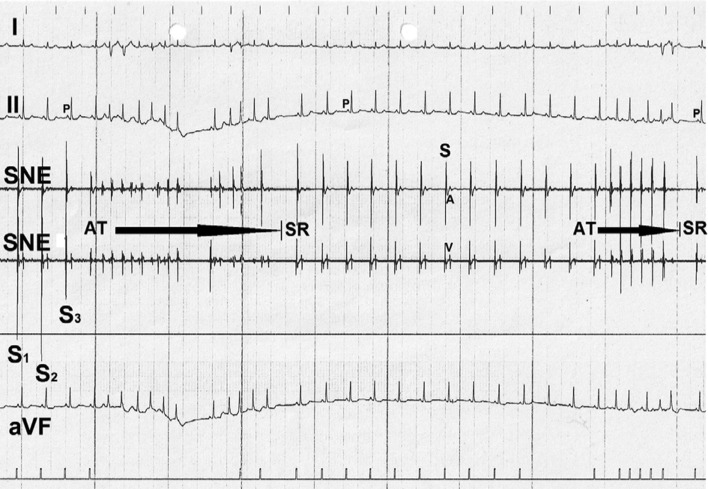
Bipolar focused sinus nodal electrograms recorded during endocardial catheter exploration of the right atrial cavity. The image shows high amplitudes of electrical potentials (S1, S2, S3) in the sinus nodal area. During manipulation of the catheter, short runs of atrial tachycardias occurred, suggesting atrial fibrillation and flutter induced presumably by mechanical irritation with the mapping catheter. I, II, aVF: surface lead electrocardiogram; P: P-waves; SR: sinus rhythm.

On the following day, under slight sedation with 15 mg of clorazepate, right heart catheterization was performed as described above. During catheter exploration of the sinus nodal area, episodes of atrial tachycardia/flutter/fibrillation occurred **(Figure 5)**. Because of the onset of a panic attack after intravenous orciprenaline during prior EP studies, the patient refused this test.

**Figure 6:**
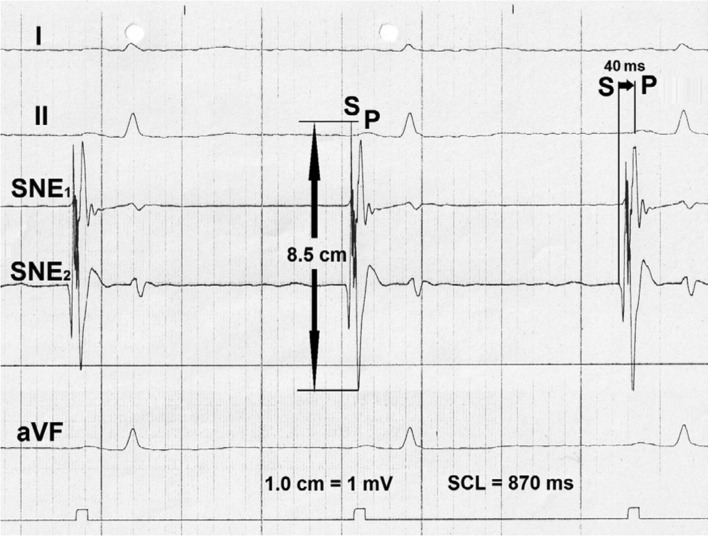
Bipolar focused sinus nodal electrograms recorded via the ELMA catheter’s pin electrodes in a patient with IST under medication with sotalol 80 mg three times daily and β-acetyl digoxin 0.2 mg. The largest (S: 8.5 cm) and earliest (SP: 40 ms) atrial electrical potential amplitudes in a small circumscribed endocardial area of the sinus nodal region can be seen. SNE: sinus nodal electrogram; I, II, aVF: surface lead electrocardiogram; SCL: sinus cycle length.

**Figure 7:**
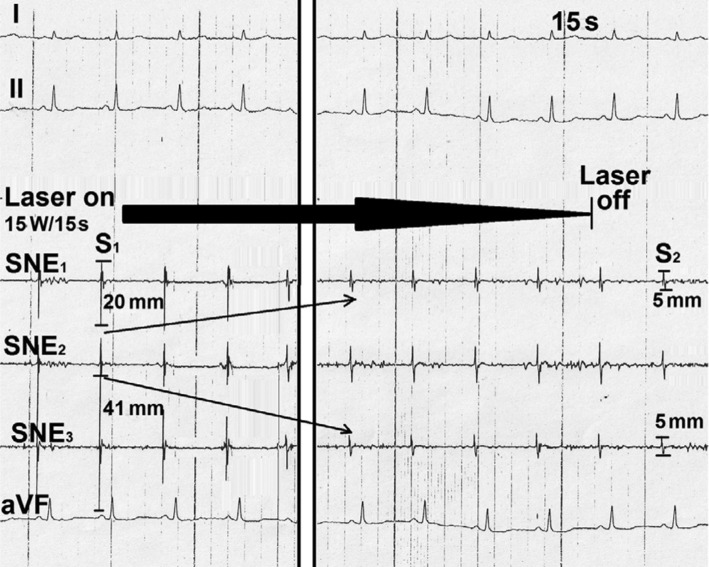
Bipolar focused sinus nodal LEGs recorded during laser application, showing a gradual abatement of electrical potential amplitudes (oblique arrows) from S1 to S2. I, II, aVF: surface lead electrocardiogram; SNE: sinus nodal electrogram.

**Figure 8:**
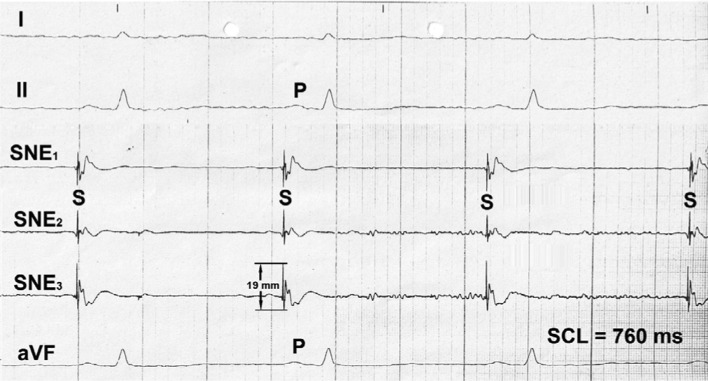
Bipolar sinus nodal electrograms showing the largest sinus nodal electrical potential amplitudes (S: maximum 1.9 mm) found by mapping the entire sinus nodal area after two laser applications. I, II, aVF: surface lead ECG; P: P-wave; SCL: sinus cycle length.

Catheter mapping allowed for localization of the earliest electrical potentials with the highest amplitudes in a very small, discreet sinus nodal area **(Figure 6)**. Following laser application aimed at that area, the height of potential amplitudes dwindled substantially and permanently. However, after replacement of the catheter in an adjacent area, again, high amplitude potentials were conspicuous in the focused LEG. With the start of a second laser impact aimed at that site, electrical potential amplitude dwindled gradually from 41.0 mm to 5.0 mm** (Figure 7)**. After the second laser impact, the maximum height of electrical potential amplitudes recorded from the sinus nodal area was ≤ 19 mm **(Figure 8)**; the use of intravenous orciprenaline 1.0 mg increased the heart rate from 78 bpm to 95 bpm with slight palpitations but without producing anxiety or panic.

**Figure 9:**
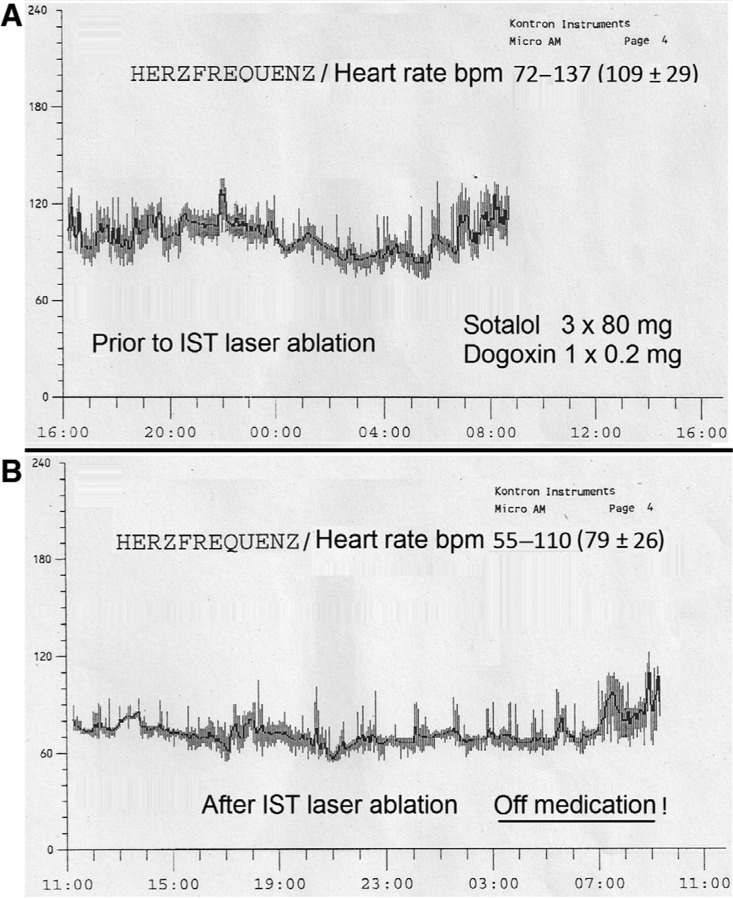
Holter monitoring registered **(A)** prior to and **(B)** after IST laser catheter ablation. A significant (p < 0.0001) decrease of the mean heart rate, from 109 bpm ± 29 bpm to 79 bpm ± 26 bpm, occurred. IST: inappropriate sinus tachycardia.

Total procedure duration time was 55 minutes, with X-ray duration time being four minutes and 42 seconds and laser application times being two instances of 15 W for 15 seconds. The ablation procedure was painless, without complication, and the patient eventually discontinued her medication. She received a Holter monitor and left the hospital six hours later. After IST ablation, her heart rate decreased from between 72 bpm and 137 bpm (109 bpm ± 29 bpm) to between 55 bpm and 110 bpm (79 bpm ± 26 bpm) (p < 0.0001) and chronotropic competence became normal **(Figure 9)**.

During a follow-up period of 4.9 years with annual controls, including a 12-lead surface ECG performed by the house doctor, the patient remained asymptomatic. Her heart rates at rest and during exercise were normal.

## Discussion

To our knowledge, this is the first published report of the successful catheter ablation of IST. The success of the laser method employed here is based on several facts. First of all, the wavelength of 1,064 nm has a low absorption in water.^7,8^ It is scattered diffusely in the myocardium and heats up the dark myoglobin selectively, creating clear-cut homogenous transmural lesions of coagulation necrosis within seconds that heal into a dense fibrous scar.^9^ Of importance is also that, by adapting laser energy settings to the thickness of the myocardial wall, collateral damages such as esophageal fistulae, lung burns, and phrenic nerve palsy can be avoided.^10^

Secondly, the open-irrigated ELMA catheter with its minielectrodes mounted at very narrow 2.0 mm interelectrode distances radially around the rim of the catheter endhole allows for the performance of high-resolution mapping by recording electrical potentials from a very small endocardial area displayed in the focused LEGs on the monitor. This improved mapping resolution cannot be achieved with the larger interelectrode distances that are more routinely used for mapping.^11–13^ We have applied the high-resolution mapping technique from the very beginning in our EP laboratories when performing EP studies in small children using 2-Fr to 4-Fr ring electrode catheters with 2.0 mm interelectrode distances.^14,15^ By using the open-irrigated ELMA catheter, focused LEG recordings are obtained without noise also during laser application.^15^ This is of crucial importance because it allows for the performance of ablation under normothermic conditions while avoiding interference with electrophysiologic monitoring principles by continuous monitoring of lesion formation. Immediate and real-time verification of the success of treatment by monitoring of local electrical potential amplitudes in the focused LEGs is extremely beneficial. In other words, electrophysiologically-guided laser catheter ablation allows for a systematic approach with simultaneous validation of initial success; a unique claim of the laser method when using the open-irrigated ELMA catheter.^16,17^

As compared with the relative uniform electrical potential amplitudes recorded during sinus nodal mapping in the healthy dogs, in the human patient with IST, a circumscribed area with high amplitude sinus nodal potentials was localized. It could not be defined whether this area was located within the sinus node or in the perinodal tissue, or even among the atrial tissue near the sinus node. However, its presence suggests a circumscribed diseased area, the culprit tissue, from which the IST was arising from. After laser applications were aimed at these sites, potential amplitudes decreased significantly and IST was ablated, indicating its causal role in the disease. As compared with in the dog experiments, where laser applications abolished local potentials, in the patient, the application of the same level of energy did not. This can be explained by the presence of a thinner myocardial wall of the dogs and/or by the thickness of the diseased atrial wall of the human patient. In addition, as compared with in the patient, in the dogs, initially, the application of the laser accelerated the heart rhythm. It can be assumed that local irritation of the sinus nodal area produced this effect and, following the ablation of a larger area, the sinus cycles lengthened.

Naturally, IST ablation as reported here is not the first laser ablation procedure performed in a patient. The reasoning for this single report is due to the rarity of the disease. Prior to this procedure, numerous laser studies were performed experimentally in our laboratory and in a series of other EP institutions in the past decades.^18^

## Conclusions

Laser modulation of the sinus node is a new intriguing alternative for the safe and effective treatment of IST. The method involves the use of a single-catheter technique and is based on substrate mapping with monitoring of local electrical potential amplitudes in the focused LEGs, without the need for sophisticated, time-consuming, and expensive mapping equipment.
